# Quercetin prevents primordial follicle loss via suppression of PI3K/Akt/Foxo3a pathway activation in cyclophosphamide-treated mice

**DOI:** 10.1186/s12958-021-00743-y

**Published:** 2021-04-23

**Authors:** Jianghui Li, Hui Long, Yanyan Cong, Hongyuan Gao, Qifeng Lyu, Sha Yu, Yanping Kuang

**Affiliations:** Department of Assisted Reproduction, Shanghai Ninth People’s Hospital Affiliated to Shanghai Jiaotong University School of Medicine, Zhizaoju Road no. 639, Huangpu District, Shanghai, People’s Republic of China

**Keywords:** Quercetin, Chemotherapy, Ovarian damage, Primordial follicle, PI3K/Akt/Foxo3a pathway

## Abstract

**Background:**

Chemotherapy improves the survival rates of patients with various cancers but often causes some adverse effects, including ovarian damage, characterised by a decrease in primordial follicle stockpiles. Recent studies have revealed that chemotherapy may stimulate the PI3K signalling pathway, thereby resulting in accelerated primordial follicle activation and a decreased ovarian reserve. Quercetin is an inhibitor of the PI3K pathway; however, its protective effects against chemotherapy-induced follicle loss in mice have not been established. In this study, the effects of quercetin in a mouse model of cyclophosphamide-induced ovarian dysfunction were investigated.

**Methods:**

C57BL/6 female mice were used for the study. Paraffin sections of mouse ovaries (*n* = 30 mice) were stained with haematoxylin and eosin for differential follicle counts. Apoptosis (*n* = 5 mice per group) was evaluated by TUNEL assay. Immunohistochemical staining for ki67 and Foxo3a (*n* = 5 mice per group) was performed to evaluate the activation of primordial follicles. The role of the PI3K signalling pathway in the ovaries (*n* = 45 mice) was assessed by western blotting.

**Results:**

Quercetin attenuated the cyclophosphamide-induced reduction in dormant primordial follicles. Analysis of the PI3K/Akt/Foxo3a pathway showed that quercetin decreased the phosphorylation of proteins that stimulate follicle activation in cyclophosphamide-induced ovaries. Furthermore, quercetin prevented cyclophosphamide-induced apoptosis in early growing follicles and early antral follicles, maintained anti-Müllerian hormone levels secreted by these follicles, and preserved the quiescence of the primordial follicle pool, as determined by intranuclear Foxo3a staining.

**Conclusions:**

Quercetin attenuates cyclophosphamide-induced follicle loss by preventing the phosphorylation of PI3K/Akt/Foxo3a pathway members and maintaining the anti-Müllerian hormone level through reduced apoptosis in growing follicles. Accordingly, quercetin is expected to improve fertility preservation and the prevention of endocrine-related side effects of chemotherapy.

**Supplementary Information:**

The online version contains supplementary material available at 10.1186/s12958-021-00743-y.

## Introduction

As an important anti-neoplastic therapeutic approach, chemotherapy improves the survival rates of patients with various cancers. However, it often has adverse effects, including ovarian damage in premenopausal women with cancer, resulting in ovarian endocrine dysfunction and an increased infertility rate [1]. The cryopreservation of gametes enables the preservation of fertility in female cancer survivors [2]. However, other outcomes of a loss of ovarian function after chemotherapy, including early menopause and its associated complications, have not been resolved [3]. Therefore, the development of a non-invasive medical prevention approach to protect ovarian function against chemotherapy-induced damage is needed.

The extent of ovarian damage largely depends on the chemotherapy regimen (i.e. drug family and dose) and patient’s age at treatment [4]. Among chemotherapy drug classes, alkylating agents (e. g. cyclophosphamide, Cy) cause the greatest damage to the ovaries, with dose-dependent effects [5]. Previous histological studies of human ovaries showed that the end-stage effects of chemotherapy are ovarian atrophy and depletion of the primordial follicle (PMF) stockpile [6]. The mechanisms underlying chemotherapy-induced PMF loss have not been fully elucidated, but may involve overactivation of PMFs [7–9].

The PMF pool is formed during foetal development in humans and is a non-renewable population representing the “ovarian reserve” in an individual [10]. To maintain the length of the female reproductive life, the majority of PMFs in humans survive in the ovary for decades in a dormant state and only few activated PMFs develop through primary and secondary stages before progressing to the antral stage [11]. A number of molecules are indispensable for the maintenance of follicular quiescence and survival [12]. Studies of genetically modified mouse models [13] have shown that forkhead transcription factor class O (Foxo) 3a maintains the dormancy of PMFs and can be inactivated by phosphorylation, leading to follicular activation. Moreover, the phosphatidylinositol 3-kinase (PI3K) signalling pathway, which regulates Foxo3a expression, regulates the survival of mammalian PMFs [12]. The phosphorylation of protein members in this pathway, such as protein kinase B (Akt/PKB), mammalian target of rapamycin (mTOR), and ribosomal protein S6 (rpS6), initiates follicle recruitment and cell growth. A balance between the positive and negative regulation of PI3K pathways is important to maintain a quiescent state in PMFs [12]. Chemotherapy may disrupt this balance, leading to a decrease in the levels of inhibitory factors or an increase in the levels of stimulatory factors, leading to the activation of dormant PMFs [7]. Based on this mechanism, the development of agents that regulate the expression of PI3K signalling pathway components may prevent the overactivation of PMF induced by chemotherapy.

Quercetin (Que), a flavonoid that is widely distributed in edible and medicinal plants, has various biological properties, without any evidence of toxicity, carcinogenicity, and genotoxicity related to its consumption [14]. Extensive research has focused on its beneficial health effects, including its preventive and therapeutic effects in cancer via interactions with multiple cancer-related pathways [15], such as the PI3K signalling pathway. In vitro, quercetin induces apoptosis and autophagy in primary effusion lymphoma cells by inhibiting PI3K/Akt/mTOR pathways [16]. In the airway of patients with chronic obstructive pulmonary disease (COPD), quercetin restores nuclear Foxo3a and reduces chemokine expression partly by modulating epidermal growth factor receptor/PI3K/Akt activity to improve lung function [17]. Thus, we hypothesised that quercetin exerts protective effects against chemotherapy-induced follicle loss by regulating the PI3K pathway. In this study, we investigated the effects of quercetin administration on follicular development and the PI3K/Akt/Foxo3a signalling pathway in a Cy-induced mouse model.

## Materials and methods

### Mice

C57BL/6 female mice aged 5–6 weeks were purchased from Jie Si Jie Laboratory Animal Co., Ltd. (Shanghai, China) and housed under specific pathogen-free conditions (22–24 °C and 12-h light/dark photoperiod), with commercial rodent diet and deionised water available ad libitum. All procedures were approved by the Ethics Committee of the Ninth People’s Hospital of Shanghai (approval No.: HKDL [2015]56).

After an acclimatisation period (5–7 d), animals were given a single intraperitoneal injection of 100 μL of phosphate-buffered saline (PBS) or an equal volume of PBS containing 75 mg/kg Cy (CAS no. 6055-19-2, Sigma-Aldrich, St. Louis, MO, USA), a dose demonstrated to destroy 50% of the PMF reserve [18]. Owing to its poor water solubility and low oral bioavailability [14], 20 [19, 20] or 40 mg/kg of quercetin (Sigma-Aldrich, Q4951) was administered intraperitoneally. Based on the overall follicle growth time in mice [21, 22], quercetin was administered daily for 14 continuous days, beginning at 1 week before Cy or PBS treatment and ending 14 days later.

The mice were sacrificed at two different time points in this study: (1) due to the activation of primordial follicles by Cy, the early growing follicles may experience a process of first rising after 3 days of Cy administration, and then falling after 7 days [7]. To observe the effect of quercetin on the follicle counts after Cy treatment clearly, the mice were euthanised by cervical dislocation to collect the ovaries at 7 days after Cy or PBS treatment for follicles counts; and (2) because the activation of the PI3K signalling pathway and the apoptosis induced by Cy are a short-term effect, the serum and ovaries were collected from the mice at 24 h after Cy or PBS treatment.

### Histological processing and follicle count

Mice (*n* = 30) were sacrificed by cervical dislocation 7 days after Cy or PBS treatment. The ovaries were dissected and fixed in 4% paraformaldehyde. Paraffin-embedded ovaries were serially sectioned at 5-μm thickness, and every fifth section was stained with haematoxylin and eosin for differential follicle counts [18]. Follicles containing oocytes with clearly visible nuclei were scored in each section as previously reported [23]. The follicle stage was classified according to the well-accepted standards established by Pedersen and Peters [7, 24]. For statistical analyses, primary follicles and secondary follicles were classified as early growing follicles (EGF). Follicle counting was performed by two experienced gynaecologic pathologists who were blinded to the group assignment. The follicle counts in each stage were determined in every fifth section and were multiplied by a correction factor of 5 as the final sum to represent the whole ovary [25, 26]. Data are presented as the number of follicles per developmental stage.

To corroborate the correct count of primordial follicles, the expression of VASA (DDX4) protein, a cytoplasmic antigen of germ cells, was detected by immunohistochemistry in ovarian sections [27, 28]. Antibody for DDX4 (1:4000; ab270534, Abcam, Cambridge, UK) was used.

### TUNEL assays

For apoptosis detection, ovarian tissues were obtained from mice (*n* = 5 mice per group) sacrificed at 24 h after Cy or PBS treatment and serially sectioned (5-μm sections). An In Situ Cell Death Detection Kit (Roche, Basel, Switzerland) was used for the in situ localisation of nuclei exhibiting DNA fragmentation by the TUNEL technique according to the manufacturer’s instructions, as previously described [29]. Sections were counterstained with haematoxylin. The number of apoptotic cells was determined by counting labelled cells from all follicle types in × 400 microscopic fields (four sections per ovary; five ovaries per group). Follicles presenting more than 5% labelled cells were considered unhealthy [26]. The apoptotic index was calculated as the fraction of unhealthy/total follicles in each class [26].

### Histochemical and immunohistochemical analyses of ovarian tissues

For immunohistochemistry analysis, ovaries were obtained from mice (*n* = 5 mice per group) sacrificed at 24 h after Cy or PBS treatment. After deparaffinisation of the ovaries in xylene and rehydration by graduated ethanol washes, endogenous peroxidase activity in the sections was inactivated by 3% (vol/vol) hydrogen peroxide in PBS. After epitope retrieval (at 98 °C for 30 min in 0.01 M citrate buffer solution, pH 6.0; Dako, Glostrup, Denmark), non-specific binding was blocked with 2% bovine serum albumin for 1 h at room temperature. The sections were incubated with primary antibody overnight at 4 °C. Antibodies for Foxo3a (1:1000; 12829S, Cell Signaling Technology, Danvers, MA, USA), Ki67 (1:400; 12202S, Cell Signaling Technology), and cleaved caspase-3 (1:200; 9661S, Cell Signaling Technology) were used. After washing, slides were incubated with biotinylated anti-rabbit IgG (Dako) for 1 h. Finally, protein signals were visualised by DAB (Dako) staining. After stopping the reaction with distilled water, the slides were counterstained with haematoxylin, dehydrated, and mounted. Images were obtained using a digital camera (Olympus, Tokyo, Japan) mounted on a conventional light microscope (Olympus) at a magnification of × 40 [26].

### Western blotting

Mice were sacrificed at 24 h after Cy or PBS treatment and the ovaries were removed, placed on ice, and homogenized in radioimmunoprecipitation assay lysis buffer supplemented with protease inhibitors [26]. After centrifugation (12,000×*g*, 4 °C for 15 min), proteins in the supernatant were measured with a BCA Protein Assay Kit (TH269580, Thermo Fisher Scientific, Waltham, MA, USA). Protein (30 μg) in loading buffer was separated by 8% sodium dodecyl sulphate-polyacrylamide gel electrophoresis. The resolved proteins were transferred onto polyvinylidene fluoride membranes (Merck Millipore, Darmstadt, Germany), and the membranes were incubated in blocking buffer (5% non-fat milk, 0.1% Tween-20 in 20 mM TBS, pH 8.0) for 1 h at room temperature and probed with specific primary antibodies overnight at 4 °C. Primary antibodies against Akt (1:1000, 4691S), phospho-Akt (Ser473) (1:1000, 4060S), mTOR (7C10) (1:1000, 2983S), phospho-mTOR (Ser2448) (1:1000, 5536S), rpS6 (5G10) (1:1000,2217S), phospho-rpS6 (Ser235/236) (1:1000, 2211S), Foxo3a (1:1000,12829S), phospho-Foxo3a (1:1000, 9464S), and β-actin (1:1000, 4970S) were all rabbit monoclonal antibodies and purchased from Cell Signaling Technology. The blot was incubated with horseradish peroxidase-conjugated goat anti-rabbit IgG (1:10000, MR-M100; MR Biotech, Shanghai, China) for 1 h at room temperature, and an enzymatic chemiluminescence kit (Pierce, Thermo Fisher Scientific) was employed for visualisation. Signals were scanned using the Amersham600 imager system (GE, Chicago, IL, USA), and the integrated light intensity for each band was quantified using ImageJ software (National Institutes of Health, Bethesda, MD, USA). These values were used to compare treatment-induced changes in the concentrations of phosphoproteins as well as to calculate the ratio of phosphorylated proteins to their non-phosphorylated forms. β-Actin expression was measured to verify equal loading. Experiments were repeated three to five times and showed similar results, and three to five ovaries were pooled for each analysis, with a total of 45 animals.

### Serum anti-Müllerian hormone (AMH) measurements

At 24 h after Cy or PBS treatment, the blood was collected by cardiac puncture at the time of sacrifice. This was performed using a 1 mL syringe and a 22-gauge needle. The needle was inserted 5 mm from the centre of the thorax towards the animal’s chin 5–10 mm deep, while holding the syringe 25–30 degrees away from the chest [30]. Next, 700–900 μl blood was collected, and the serum was separated by centrifugation at 1000×*g* for 15 min at 4 °C and stored at − 80 °C until further analysis. Serum AMH concentrations were quantified using a Mouse Mullerian-Inhibiting Factor ELISA Kit (Signalway Antibody, College Park, MD, USA) according to the manufacturer’s instruction, as described in a previous study [31] (*n* = 5 mice per group). The coefficient of variation (CV) for the AMH assay is as follows: intra-assay CV: ≤6.5%; inter-assay CV: ≤10.2%.

### Statistical analyses

Results are expressed as the means ± standard error of the mean (SEM) and were analysed using SPSS software (version 24, SPSS Inc., Chicago, IL, USA). Differences between groups were evaluated by one-way analysis of variance (ANOVA) followed by Tukey’s tests in all cases. Shapiro–Wilk test was used for normality before ANOVA. *P* < 0.05 was considered to indicate statistically significant result.

## Results

### Quercetin reduces cy-induced PMF loss

**Fig. 1:**
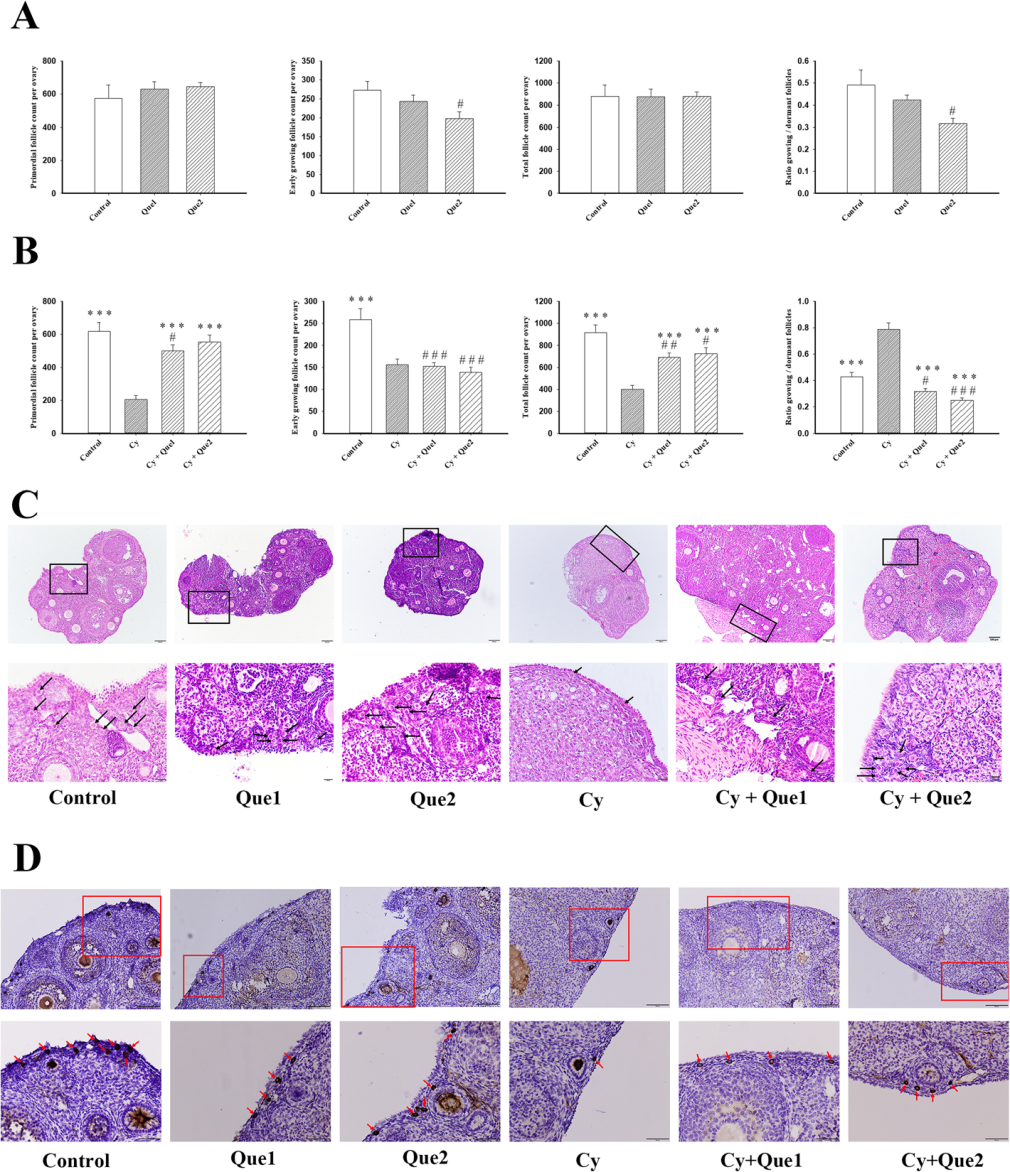
Quercetin reduces Cy-induced PMF loss. **a**-**b** Effects of PBS alone, quercetin alone, Cy alone, or Cy with quercetin on different stages of follicle development (*n* = 5 mice/group). Data are expressed as the means ± SEM. One-way ANOVA followed by Tukey’s tests was used for comparisons (***P < 0.001 compared with the Cy group; #*P* < 0.05, ##*P* < 0.01, ###*P* < 0.001 compared with the control group). **c** Representative images of haematoxylin and eosin staining ofthe ovarian histological sections from all experimental groups. Scale bars represent 100 μm (upper panel in each group) and 20 μm (lower panel in each group). Inset boxes denote areas shown in higher magnification to the below. Black arrows indicate the primordial follicles. **d** Representative images of DDX4-immunostained ovarian histological sections from all experimental groups. Inset boxes denote areas shown in higher magnification to the below. Red arrows indicate primordial follicles. Scale bars represent 500 μm

First, the effects of quercetin alone on different stages of follicle development were investigated. The total number of follicles after quercetin administration did not differ from that in the control group. However, as the quercetin dosage was increased, the number of PMFs increased slightly (but not significantly) and the EGF count decreased gradually, especially in the high-dose quercetin group (*P* < 0.05 compared with control mice, Fig. 1a). The ratio of EGF to PMF was lower in the high-dose quercetin group than in the control group (*P* < 0.05 compared with control mice, Fig. 1a). These results suggest that quercetin has the potential to inhibit the transition from PMFs to the next developmental stage.

To determine the impact of quercetin on Cy-induced follicle loss, adult female mice were treated with quercetin (20 or 40 mg/kg per mouse) daily for 14 continuous days, beginning at 1 week before Cy treatment and ending 14 days later. Histological analysis showed that Cy-treated ovaries contained fewer follicles than did control ones, presenting cortical fibrosis and altered stromal cells, while ovaries cotreated with Cy and quercetin contained a similar number of follicles to healthy control ovaries (Fig. 1c and d). Furthermore, mice treated with Cy showed dramatic reductions in the numbers of PMFs, EGFs, and total follicles compared with those in control animals (*P* < 0.001, Fig. 1b). Quercetin significantly attenuated the Cy-induced decrease in the number of PMFs, especially at a concentration of 40 mg/kg, which resulted in the maintenance of an ovarian reserve equivalent to that of untreated controls (616.56 ± 55.41 vs. 554.00 ± 41.60, *P* = 0.275). However, neither dosage of quercetin prevented the loss of EGFs caused by Cy (Fig. 1b). Accordingly, the ratio of EGFs to PMFs was low in mice after Cy and quercetin co-treatment, even lower than that in the control. Finally, the follicle sum was partially rescued in the Cy and quercetin co-treatment group, which showed higher values than did the Cy group (*P* < 0.001), but did not reach the level in the control group (Fig. 1b). Therefore, the quantification of follicles at different stages revealed that quercetin at both dosages prevented the loss of PMFs induced by Cy, but did not protect against Cy-induced damage to EGFs.

### Quercetin prevents apoptosis in EGF and the decreased AMH induced by cy

**Fig. 2:**
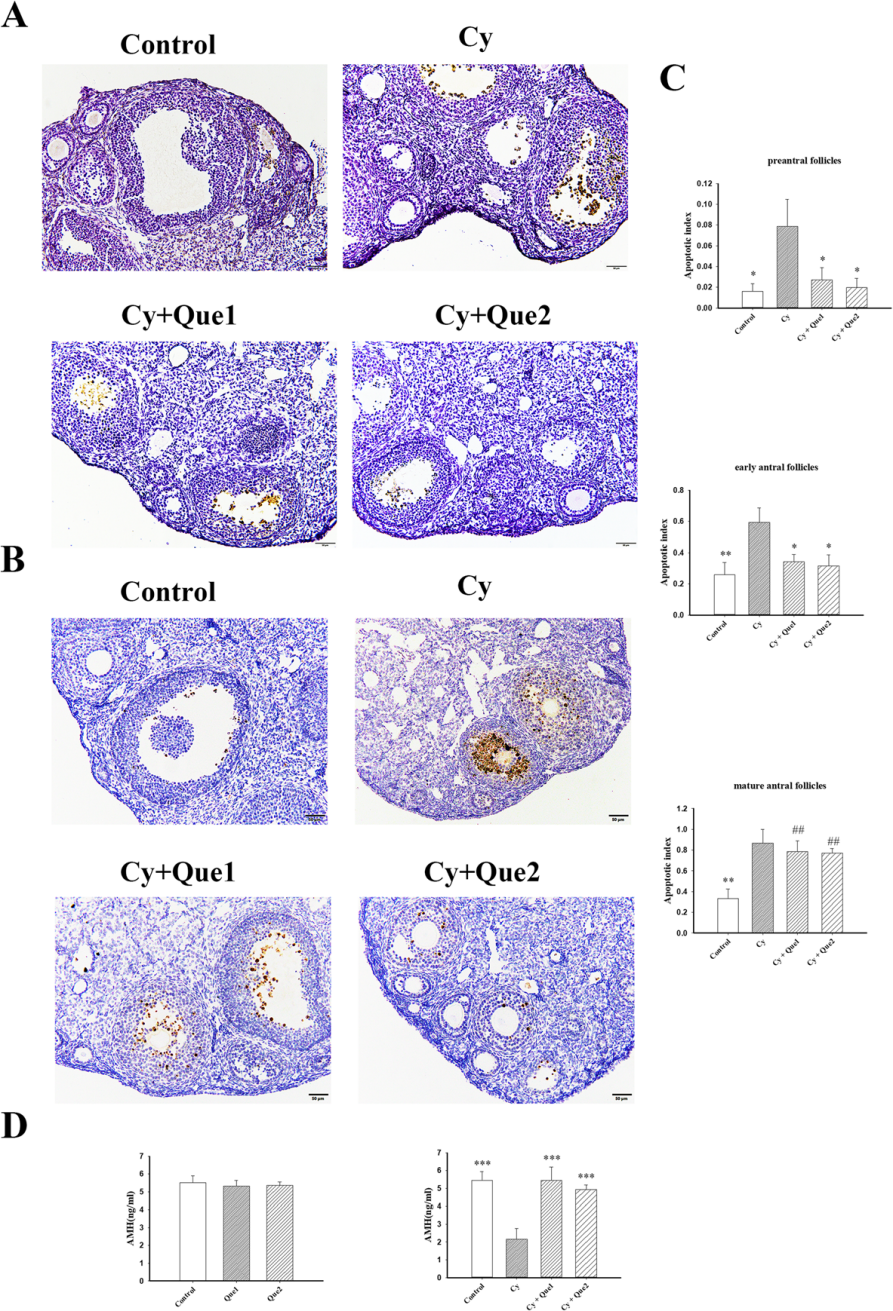
Quercetin prevents apoptosis in growing follicles and decreases AMH induced by Cy. **a**-**b** Representative images of mouse ovaries stained with TUNEL (**a**) and cleaved caspase-3 antibody (**b**) are shown for each treatment condition. Bars represent 50 μm. **c** TUNEL-based quantification of the apoptotic index per follicle class calculated as the fraction of apoptotic/total follicles for each type (*n* = 5 mice/group, *P < 0.05, **P < 0.01, compared with the Cy group; ## *P* < 0.01 compared with the control group). Primordial follicles did not present TUNEL-positive cells in any experimental group. One-way ANOVA followed by Tukey’s tests was used for comparisons. **d** AMH plasma concentrations were measured at 24 h after administration of PBS or Cy with or without quercetin (*n* = 5 mice/group). Data represent the means ± SEM. One-way ANOVA followed by Tukey’s tests was used for comparisons (***P < 0.001 compared with the Cy group)

Apoptosis is a possible mechanism underlying ovarian damage induced by chemotherapy [32–36]; accordingly, we explored whether the preventive effects of quercetin on Cy-induced PMF loss were mediated by the inhibition of apoptosis. However, neither cleaved caspase-3 staining nor TUNEL staining showed any evidence of apoptosis in PMFs after Cy treatment with or without quercetin. As follicle development progressed, the apoptosis of granulosa cells induced by Cy gradually increased, and the addition of quercetin may attenuate this increase slightly (Fig. 2a and b).

To further verify the anti-apoptotic effect of quercetin in growing follicles, the apoptotic index (TUNEL-positive follicles/total follicles) was quantified based on TUNEL sections (Fig. 2c). Cy treatment increased the apoptotic index of all growing follicles compared with that of control ovaries, and quercetin coadministration inhibited apoptosis in the follicles at the preantral and early antral stages, as evidenced by the significantly lower apoptotic indexes for follicles at these two stages after quercetin cotreatment than for those after Cy treatment alone (*P* < 0.05), similar to the value in controls (Fig. 2c). However, this protective effect of quercetin was not obvious in granulosa cells of mature antral follicles.

AMH is expressed by granulosa cells and is produced by growing follicles from the primary stage of development until selection for dominance [37]. A study of *Amh*^*−/−*^ mice showed that AMH facilitates the maintenance of PMFs in a dormant state [38]. As an indirect indicator of the follicle reserve, AMH was measured at 24 h after Cy administration. As shown in Fig. 2d, the AMH concentration was significantly lower after Cy treatment (2.2 ng/mL) than in controls (5.4 ng/mL, *P* < 0.001); however, the coadministration of Cy and quercetin resulted in a similar concentration of AMH to that in the control. Quercetin alone did not alter the concentration of serum AMH.

These results indicate that Cy-induced PMF loss may not directly induce apoptosis in PMFs, but may have an indirect effect by acutely reducing growing follicles and AMH levels, resulting in increased recruitment of PMFs to the growing pool. Quercetin cotreatment may inhibit apoptosis induced by Cy in growing follicles and maintain the levels of AMH secreted by these follicles, thus maintaining the dormant state.

### Quercetin inhibits cy-induced PI3K/Akt/Foxo3a signalling pathway activation

Recent studies have indicated that Cy-induced loss of the ovarian reserve is related to the acceleration of PMF activation via activation of the PI3K signalling pathway [7]. Therefore, we investigated the effects of quercetin on the PI3K signalling pathway in the ovaries after Cy treatment.

**Fig. 3:**
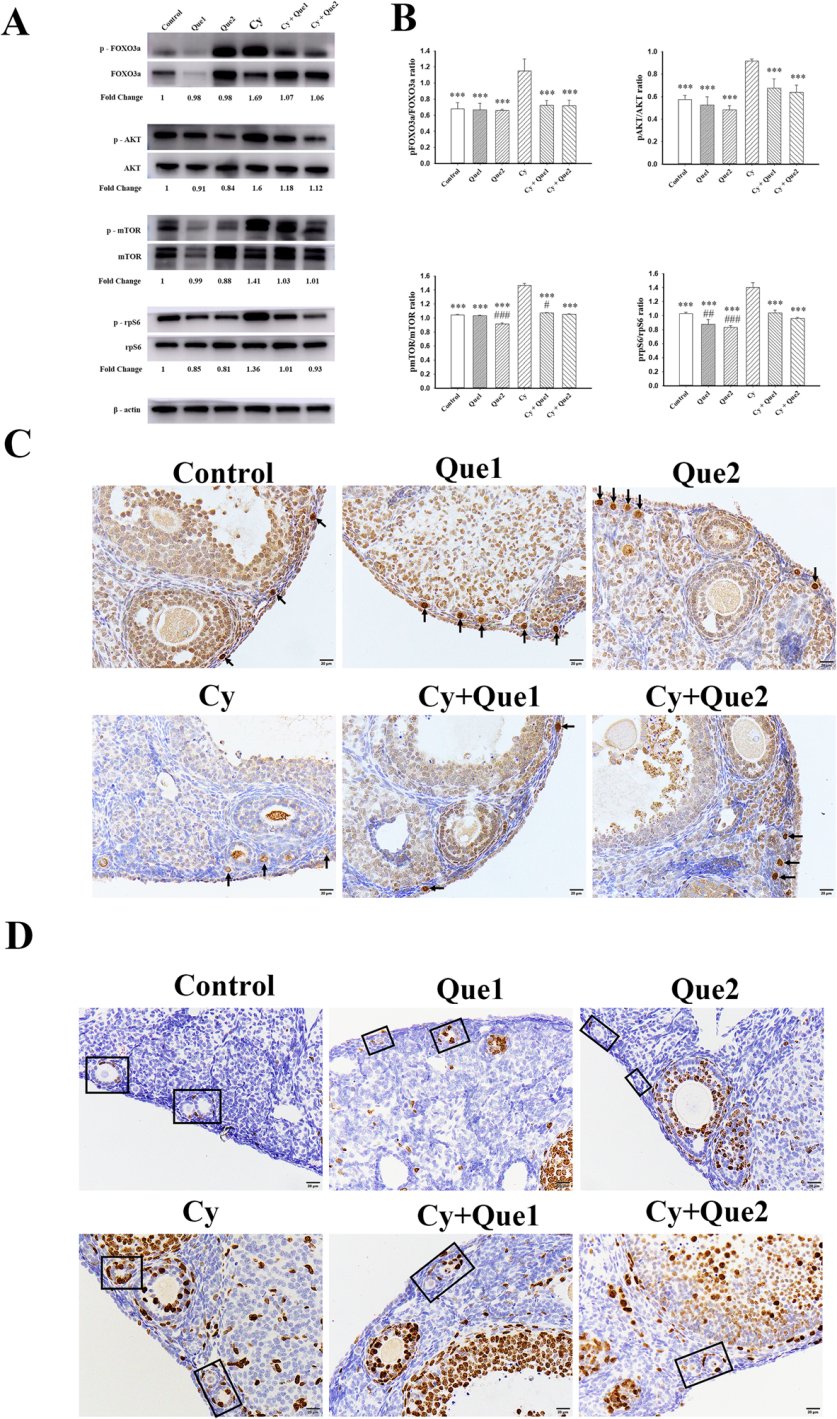
Quercetin reduces the Cy-induced phosphorylation of PI3K/Akt/Foxo3a pathway proteins in the ovary. **a** Western blot analyses were used to evaluate ovaries from 8-week-old mice removed at 24 h after a single dose of Cy (75 mg/kg) or an equivalent volume of PBS with or without quercetin (Que1: 20 mg/kg; Que2: 40 mg/kg). Comparisons of the concentrations of phosphorylated and total Foxo3a, Akt, mTOR, and rpS6, using β-actin as a loading control. Fold-change values for ratios for each protein are listed below each set of bands. **b** Ratios of phosphorylated/total protein are summarised in a bar graph. Experiments were repeated three to five times with similar results; three to five ovaries were pooled per result, with a total of 45 animals. The data are presented as the mean values ± SEM. (***P < 0.001 compared with the Cy group; # *P* < 0.05, ## *P* < 0.01, ### *P* < 0.001 compared with the control group). One-way ANOVA followed by Tukey’s tests was used for statistical analyses. **c**-**d** Immunohistochemical staining for Foxo3a and Ki67 demonstrates that quercetin inhibited Cy-induced primordial follicle overactivity and short-term growth. A representative image from each group is shown. **c** immunohistochemical staining for Foxo3a shows the localisation of Foxo3a in each group. Arrows indicate primordial follicles. **d** Ki67 staining of ovary tissues was used to evaluate cell proliferation. Black frames indicate primary follicles. Scale bars, 20 μm

Foxo3a, a downstream effector of the PI3K signalling pathway, is highly expressed in the nuclei of oocytes of PMFs and is important for the maintenance of PMFs in the dormant state [13]. Its nuclear export results in the activation of PMFs and initiation of development [39]. In this study, Foxo3a expression was not detected by immunohistochemical staining in the PMF population after Cy administration; however, intense staining was observed in the quercetin cotreatment group, similar to that in the control group (Fig. 3c). Western blotting further showed that the ratio of phosphorylated Foxo3a to the non-phosphorylated form was 1.69-fold higher in Cy-treated ovaries than in the control group, with recovery to control levels after quercetin co-administration. However, quercetin alone did not significantly affect the expression of Foxo3a (Fig. 3a and b).

Moreover, following their activation, PMFs enter the stage of growth and granulosa cells begin to proliferate. Immunostaining for the proliferation marker Ki67 in control ovaries showed that granulosa cell proliferation was obvious in large growing follicles, with only occasional staining in small activated follicles. Interestingly, substantial Ki67 staining was observed in granulosa cells in transitional primordial and primary follicles from ovaries removed at 24 h after Cy administration, with fewer positive cells in the quercetin cotreatment group than in the Cy-treated group (Fig. 3d).

In addition to the suppressor Foxo3a, the key activation proteins Akt, mTOR, and rpS6 were detected. Cy-treated ovaries showed an increase in the phosphorylated forms of these proteins (1.3–1.6-fold increases compared to control group level, Fig. 3a and b). These increased levels of phosphorylation caused by Cy treatment were attenuated by cotreatment with quercetin, which also suppressed the phosphorylation of mTOR and rpS6 alone, particularly when administered at a high dosage.

These results suggest that quercetin prevents activation of the PI3K signalling pathway induced by Cy to preserve the ovarian reserve.

## Discussion

The precise mechanisms of chemotherapy-induced ovarian damage remain unclear. Recently, the ‘burn-out effect’ [7–9] or direct DNA damage of the follicle reserve [36] has been suggested as the possible mechanisms of chemotherapy-induced ovarian damage. Chemotherapy may disrupt the function of the PI3K/PTEN/Akt signalling pathway responsible for follicle quiescence, thereby directly inducing PMF overactivation and depletion of follicle reserve [7]. But current researches show that oocytes leave their follicular quiescence to initiate the DNA damage signalling pathway leads to oocyte apoptosis [40] and oocyte damage leading to PUMA-dependent apoptotic cell death being the predominant process involved in primordial follicle loss after treatment with Cy [41]. However, herein, apoptosis in the PMFs was not observed 24 h upon Cy treatment. In fact, oocytes are capable of efficient DNA repair in response to the inhibition of the apoptotic pathway in the ovary [42]. This contrasting result may be related to the time of the observation, as is possible that oocytes may have achieved complete DNA repair at this time point; nonetheless, this needs to be further confirmed. Furthermore, with the growth and development of follicles, granulosa cells proliferate vigorously and are, thus, more vulnerable to the damaging effects of Cy. Hence, Cy can promote massive apoptosis in growing follicles, thereby reducing the secretion of inhibitory factors and indirectly recruit PMFs to the growing pool [7].

In this study, the effects of quercetin, an inhibitor of PI3K [15], on Cy-induced follicle loss were evaluated. The administration of quercetin alone slightly and dose-dependently increased the number of PMFs and decreased the number of EGFs, particularly at a high dose. Cotreatment with quercetin and Cy rescued the number of dormant PMFs to that in control ovaries. Western blotting showed that although administration of Cy increased the phosphorylation levels of Akt, mTOR, and rpS6, which are involved in the regulation of PMF activation, quercetin decreased their levels, and the phosphorylation of Foxo3a, an indicator of PMF activation, was also decreased after quercetin administration. These results collectively indicate that quercetin may inhibit the transformation from PMFs to primary follicles induced by Cy by inhibiting the PI3K signalling pathway. However, the PI3K signalling pathway is important for various physiological processes [43]. Further studies are thus needed to determine whether quercetin negatively regulates the growth and development of normal cells via inhibition of the PI3K signalling pathway.

In addition to the direct mechanism described above, the PMF pool can be reduced indirectly via the loss of activated, growing follicles [4]. Our results revealed that chemotherapy promotes apoptosis in granulosa cells in growing follicles, with more mature follicles showing a higher rate of apoptosis. Accordingly, the suppression of follicular activation by AMH from granulosa cells of growing follicles is decreased, leading to the accelerated depletion of the PMF reserve [44]. As a result, PMF growth is initiated to replace damaged growing follicles. In this study, Foxo3a staining showed that PMFs are activated, and Ki67 staining showed that the proliferation of granulosa cells in transitional primordial and primary follicles is activated after chemotherapy. However, quercetin coadministration protected follicles at the preantral and early antral stages from apoptosis and maintained AMH levels, leading to preservation of the quiescence of the ovarian reserve. We speculate that this phenomenon is also related to the inhibitory effects of quercetin on the PI3K signalling pathway and its anti-proliferation effects that weaken the sensitivity of preantral follicles and early antral follicles to Cy-induced damage. However, quercetin had no protective effects on granulosa cells of mature antral follicles against damage induced by Cy; thus, the combination of quercetin with additional anti-apoptotic compounds should be considered in future research.

Quercetin has a variety of biological functions, such as anti-inflammatory, anti-oxidant, and anti-cancer effects [45], and a reasonable dosage is important for its biological activity [46]. Numerous in vitro studies have reported that quercetin promotes apoptosis at high dosages and shows anti-oxidant effects at low concentrations [15]. A previous study demonstrated that intraperitoneal injection of 20 mg/kg quercetin has protective effects against ovarian toxicity and POF induced by Cy, without compromising its antitumor effect [19]. However, in a xenograft tumour model, quercetin at 20 mg/kg may attenuate the therapeutic effects of chemotherapeutic drugs in ovarian cancer cells by reducing reactive oxygen species-induced damage [20]. Therefore, both 20 mg/kg and a relatively high dose of 40 mg/kg were chosen for this study. Both dosages of quercetin inhibited the activation of PMFs induced by Cy, specifically at the high dosage. Moreover, the overall follicle growth time in mice is 17 to 19 days [21] and the duration from preantral follicles to ovulatory follicles is about 12 days [22]; accordingly, the transformation from PMFs to primary follicles may occur within 7 days. Hence, quercetin can cover this process by daily administration for 1 week before Cy administration. However, because of the follicle counts observed at 7 days after Cy administration and the short biological half-life of quercetin [14], we continued quercetin administration for 7 days after Cy injection; thus, quercetin was administered for 14 continuous days, beginning at 1 week before Cy or PBS treatment and ending 14 days later, to maximise the protective effect of quercetin against Cy-induced damage to follicles.

## Conclusions

**Fig. 4:**
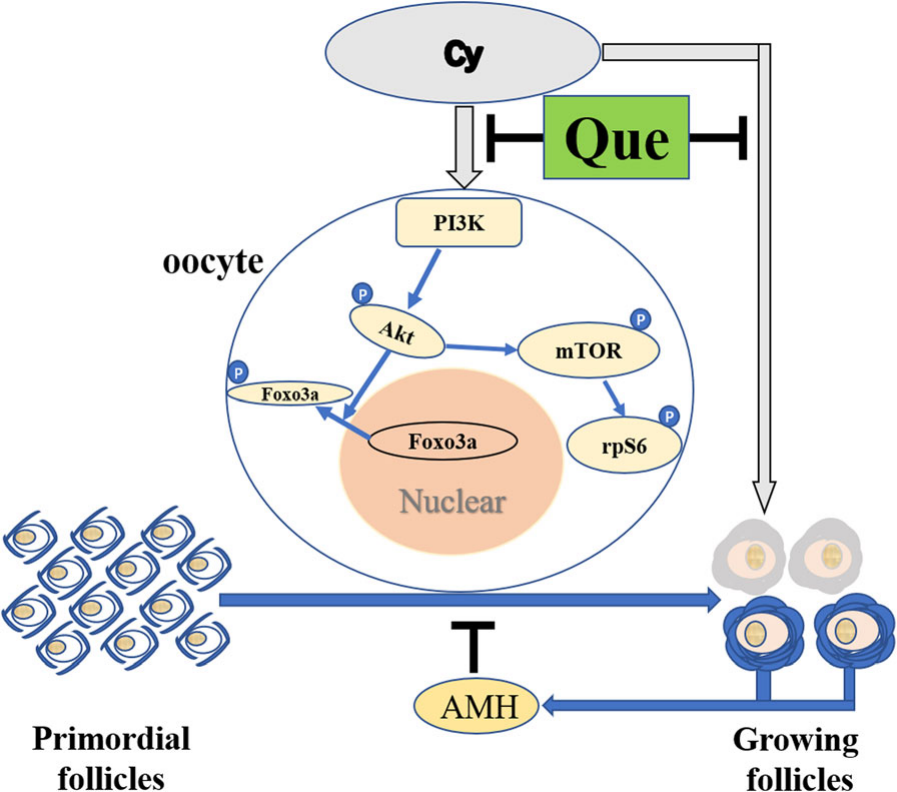
Quercetin inhibits overactivation of dormant PMFs induced by Cy. In normal circumstances, the dormancy or activation of primordial follicles was regulated by PI3K signalling pathway and the inhibitor factors secreted by growing follicles, such as AMH. Cyclophosphamide disrupts the balance of the PI3K signalling pathway and decreases AMH level by inducing apoptosis in growing follicles, resulting in the overactivation of the primordial follicles and the burnout of the reserve. Coadministration of quercetin and Cy prevents the phosphorylation of PI3K/Akt/Foxo3a pathway members and maintains AMH level through reduced apoptosis in growing follicles to restore the ovary to a state of equilibrium

In summary, this in vivo mouse study provides the first evidence that quercetin inhibits the Cy-induced overactivation of dormant PMFs via regulation of the PI3K/Akt/Foxo3a signals and maintaining AMH level through reduced apoptosis in growing follicles, thereby restoring the ovary to a state of equilibrium (Fig. 4). Because of its ability to prevent the loss of follicles induced by chemotherapy, quercetin is expected to be a non-invasive medical approach to prevent infertility as well as endocrine-related side effects in female cancer survivors. The results of our research also expand our understanding of the applications of quercetin.

## Supplementary Information

**Additional file 1: Figure S1.** The apoptosis of granulosa cells in growing follicles at 7 days after PBS or Cy treatment with or without quercetin. (A) Representative images of mouse ovaries stained with TUNEL are shown for each treatment condition. Bars represent 100 μm. (B) TUNEL-based quantification of the apoptotic index per follicle class calculated as fraction apoptotic/total follicles for each type. *n* = 5 mice. (At 24 h after PBS or Cy treatment with or without quercetin, *P < 0.05, **P < 0.01, compared with the Cy group; ## *P* < 0.01 compared with the control group; At 7 days after PBS or Cy treatment with or without quercetin, & *P* < 0.05, && *P* < 0.01, &&& *P* < 0.001 compared with the control group). One-way ANOVA followed by Tukey’s tests was used for comparisons.

**Additional file 2: Figure S2.** Protective effect of quercetin on the number of primordial follicles after Cy treatment (*n* = 3 mice/group). Data are expressed as the means ± SEM. One-way ANOVA followed by Tukey’s tests was used for comparisons (***P < 0.001, **P < 0.01 compared with the Cy group; ##*P* < 0.01 compared with the control group).

## Data Availability

All data generated through this study are included in this article.

## Abbreviations

Cy: Cyclophosphamide
PMF: Primordial follicle
Foxo: Forkhead transcription factor class O
PI3K: Phosphatidylinositol 3 - kinase
Akt/PKB: Protein kinase B
mTOR: mammalian target of rapamycin
rpS6: ribosomal protein S6
Que: Quercetin
EGF: Early growing follicles
AMH: Anti-Müllerian hormone
POF: Premature ovarian failure
