# Supplementary figures and images for: Quercetin prevents primordial follicle loss via suppression of PI3K/Akt/Foxo3a pathway activation in cyclophosphamide-treated mice
Source: Reprod Biol Endocrinol. 2021 Apr 23;19:63. doi: 10.1186/s12958-021-00743-y (PMC8063466; doi:10.1186/s12958-021-00743-y)

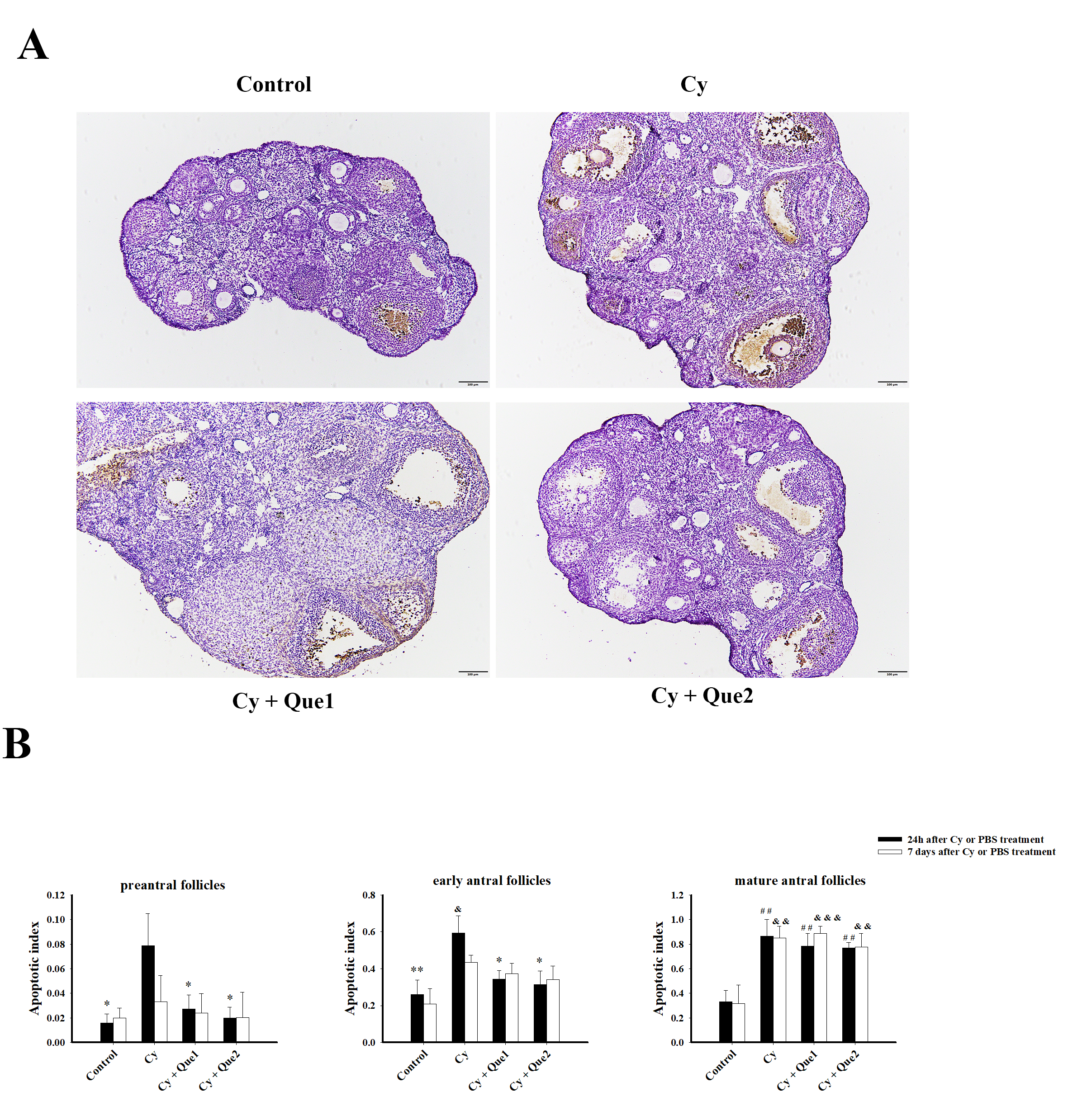

Supplement: Supplementary file 1 — Additional file 1: Figure S1. The apoptosis of granulosa cells in growing follicles at 7 days after PBS or Cy treatment with or without quercetin. (A) Representative images of mouse ovaries stained with TUNEL are shown for each treatment condition. Bars represent 100 μm. (B) TUNEL-based quantification of the apoptotic index per follicle class calculated as fraction apoptotic/total follicles for each type. n = 5 mice. (At 24 h after PBS or Cy treatment with or without quercetin, *P < 0.05, **P < 0.01, compared with the Cy group; ## P < 0.01 compared with the control group; At 7 days after PBS or Cy treatment with or without quercetin, & P < 0.05, && P < 0.01, &&& P < 0.001 compared with the control group). One-way ANOVA followed by Tukey’s tests was used for comparisons. [file 12958_2021_743_MOESM1_ESM.tif]

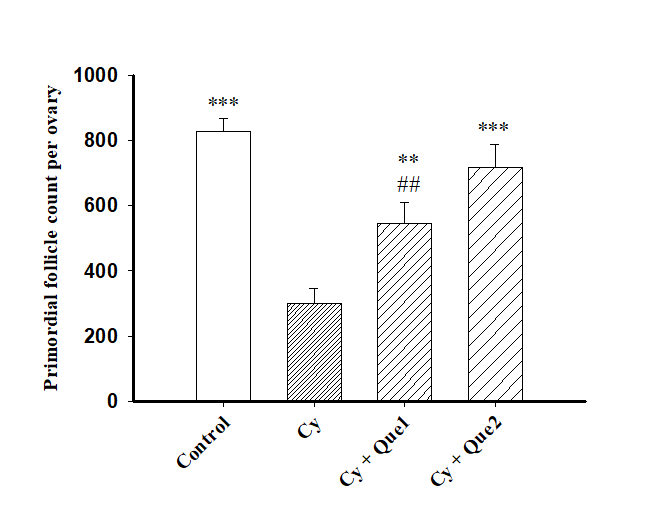

Supplement: Supplementary file 2 — Additional file 2: Figure S2. Protective effect of quercetin on the number of primordial follicles after Cy treatment (n = 3 mice/group). Data are expressed as the means ± SEM. One-way ANOVA followed by Tukey’s tests was used for comparisons (***P < 0.001, **P < 0.01 compared with the Cy group; ##P < 0.01 compared with the control group). [file 12958_2021_743_MOESM2_ESM.tif]
